# Development of a prognostic prediction model based on a combined multi-omics analysis of head and neck squamous cell carcinoma cell pyroptosis-related genes

**DOI:** 10.3389/fgene.2022.981222

**Published:** 2022-09-29

**Authors:** Bin Chen, Yuanbo Luo, Xueran Kang, Yuxing Sun, Chenyan Jiang, Bin Yi, Xiaojun Yan, Yisheng Chen, Runjie Shi

**Affiliations:** ^1^ Department of Otorhinolaryngology Head and Neck Surgery, Ninth People’s Hospital, Shanghai Jiaotong University, School of Medicine, Shanghai, China; ^2^ Ear Institute, School of Medicine, Shanghai Jiaotong University, Shanghai, China; ^3^ Shanghai Key Laboratory of Translational Medicine on Ear and Nose Diseases, Shanghai, China; ^4^ Department of Sports Medicine, Huashan Hospital, Fudan University, Shanghai, China

**Keywords:** prognostic model, nasopharyngeal carcinoma (NPC), pyroptosis, immune infiltration, head and neck squamous cell carcinoma (HNSCC)

## Abstract

This study aimed to understand the prognosis of patients with head and neck squamous cell carcinoma (HNSCC) and to develop and validate a prognostic model for HNSCC based on pyroptosis-associated genes (PAGs) in nasopharyngeal carcinoma. The Cancer Genome Atlas database was used to identify differentially expressed PAGs. These genes were analyzed using the Kyoto Encyclopedia of Genes and Genomes functional annotation analyses and Gene Ontology analyses. The NLR family pyrin domain containing 1 (*NLRP1*) gene, charged multivesicular body protein 7 (*CHMP7*) gene, and cytochrome C (*CYCS*) gene were used to create a prognostic model for HNSCC. The results of the Kaplan-Meier (K-M) and Cox regression analyses indicated that the developed model served as an independent risk factor for HNSCC. According to the K-M analysis, the overall survival of high-risk patients was lower than that of low-risk patients. The hazard ratios corresponding to the risk scores determined using the multivariate and univariate Cox regression analyses were 1.646 (95% confidence interval (CI): 1.189–2.278) and 1.724 (95% CI: 1.294–2.298), respectively, and the area under the receiver operator characteristic curve was 0.621. The potential mechanisms associated with the functions of the identified genes were then identified, and the tumor microenvironment and levels of immune cell infiltration achieved were analyzed. The immune infiltration analysis revealed differences in the distribution of Th cells, tumor-infiltrating lymphocytes, regulatory T cells, follicular helper T cells, adipose-derived cells, interdigitating dendritic cells, CD8^+^ T cells, and B cells. However, validating bioinformatics analyses through biological experiments is still recommended. This study developed a prognostic model for HNSCC that included *NLRP1*, *CHMP7*, and *CYCS.*

## Introduction

Head and neck squamous cell carcinoma (HNSCC) is a highly heterogeneous malignancy of various anatomical sites in the upper respiratory and digestive tracts. The sites of its origin are the paranasal sinuses, nasal cavity, oropharynx, oral cavity, and larynx. HNSCC accounts for 90% of all HNSCC cases (Bray et al., 2018; Ferlay et al., 2019). Each year, approximately 450,000 deaths and 890,000 new cases of HNSCC are recorded worldwide (Bray et al., 2018; Ferlay et al., 2019). HNSCC causes include smoking, alcohol consumption, and viral infections (Stein et al., 2015; Johnson et al., 2020). More than half the patients with HNSCC are diagnosed at an advanced stage of HNCC due to the lack of effective clinical risk assessment tools and early-stage diagnostic resources, resulting in a low survival rate (34.9%) (Chauhan et al., 2015). Currently, treatment options are selected, and the overall survival (OS) of HNSCC patients is primarily determined using the tumor–lymph node–metastasis (TNM) staging system developed by the American Joint Committee on Cancer (AJCC) (Amin et al., 2017; Keung and Gershenwald, 2018). Though this system is simple to implement and useful in a wide range of fields, it only considers tumor-related anatomical information and ignores biological heterogeneity. As a result, the ability to predict risk and assess the prognosis for patients with HNSCC is limited. Therefore, developing a novel, valid, and robust risk prediction and prognosis-assessment model is critical to improving the risk prediction accuracy and individualized treatment process.

Intracellular genes regulate cell death (including apoptosis, necroptosis, ferroptosis, pyroptosis, necrosis, autophagy, and others), which significantly impacts the process of immune system development (Fink and Cookson, 2005; Bedoui et al., 2020; Chen et al., 2021; Shi et al., 2021). Pyroptosis, a novel form of caspase-1-mediated programmed cell death, is characterized by the rapid rupture of the plasma membrane. Following the rupture, cellular contents and pro-inflammatory substances such as interleukins are released. This triggers an inflammatory cascade response, resulting in cellular damage. The process has a significant impact on tumor progression, including tumor proliferation, metastasis, and invasion (He et al., 2016; Tsuchiya, 2020). Pyroptosis induces the onset and progression of various diseases, including hepatocellular carcinoma, leukemia, lung cancer, breast cancer, gastric cancer, cervical cancer, and colorectal cancer (He et al., 2016). The dual role of pyroptosis significantly affects tumor pathogenesis. During pyroptosis, multiple signals are generated, and inflammatory mediators are released. The generation of these signals and the release of these mediators have an impact on tumorigenesis and resistance to chemotherapeutic agents. The high expression level of the pyroptosis effector gasdermin D promotes the process of tumor metastasis. For example, it is associated with a poor prognosis in patients with lung adenocarcinoma (Gao et al., 2018). Moreover, the increased susceptibility of cells to caspase-3-dependent signaling pathways that trigger pyroptosis can increase melanoma cells’ resistance to etoposide (Lage et al., 2001). Pyroptosis, on the other hand, may inhibit tumor onset and progression (Fiddian-Green and Silen, 1975; Yu et al., 2021). The expression of the pyroptosis effector gasdermin E accelerates tumor cell phagocytosis. The action of the tumor-associated macrophages mediates the process. As a result, the number of CD8^+^ T lymphocytes and tumor-infiltrating natural killer lymphocytes increases (Ding et al., 2016). CD8^+^ T lymphocytes and tumor-infiltrating natural killer lymphocytes have also shown improved function. Additionally, downregulation of the oncogene LncRNA–XIST inhibits the progression of non-small cell lung cancer. The activation of the miR-335/SOD2/ROS cascade-related pyroptosis process results in the downregulation of the oncogene (Liu et al., 2019). However, more research into the link between HNSCC and pyroptosis is needed.

Researchers recently discovered that pyroptosis is crucial in developing nasopharyngeal carcinoma (NPC) (Cai et al., 2021; Xia et al., 2021). NPC arises from epithelial cells in the nasopharynx, and squamous carcinoma is the most common type (Chen et al., 2019). Exploring the relationship between NPC and pyroptosis-associated genes (PAGs) could aid in the understanding of HNSCC. Basic medical research can benefit from bioinformatics as it can provide information at multiple levels and aspects about molecular mechanisms of disease (Holtsträter et al., 2020; Lin et al., 2021; Chen et al., 2022; Luo et al., 2022; Sun et al., 2022; Yan et al., 2022). Biomarkers related to PAGs for NPC-related bioinformatics can provide effective treatment for HNSCC. This study combined genomic, transcriptomic, proteomic, metabolomic, and immunomics data to explore the microenvironmental composition of head and neck tumors and identify indicators associated with patient prognosis. A prognostic model for HNSCC was developed and validated based on PAGs in NPC.

## Methods

### Data download and pre-processing

Nasopharyngeal carcinoma (NPC)-related gene expression profiles was obtained from the Gene Expression Omnibus (GEO) database. The keyword “nasopharyngeal carcinoma” was selected to obtain three eligible mRNA microarray datasets (GSE12452, GSE53819, and GSE64634). GSE12452 and GSE64634 were from the GPL570 platform, whereas GSE53819 was based on GPL6480. 61 NPC samples and 32 normal samples were obtained and normalized using R 4. 0. 3 software. In cases where a single gene was associated with multiple probes, the value for the expression of that gene was set to the average expression value corresponding to the multiple probes. Additionally, a batch correction was performed using the ComBat function in the sva package to eliminate the effect of different biological companies, researchers, and experimental batches on the results. Raw ribonucleic acid (RNA) sequencing data and clinical information were also downloaded from the TCGA database. Information on the survival time, age, survival status, clinical stage, gender, tumor grade, TNM staging, and pathological stage was obtained.

### Identification of pyroptosis-related long non-coding RNAs

A total of 52 pyroptosis-related lncRNAs were obtained from literature reports (Broz et al., 2020; Wang et al., 2020; Zhou et al., 2020; Tan et al., 2021). Subsequently, the co-expression for lncRNAs and PAGs was studied using the Person correlation analysis method and the limma package in R was used to study the pyroptosis-related lncRNAs (correlation coefficient ≥0. 4; *p* < 0. 001).

### Expression analysis of *BAK1, NLRP1, CHMP7, RIPK1*

We used the Gene Expression Profiling Interactive Analysis (GEPIA) 2. 0 (http://gepia.cancer-pku.cn/), which integrates gene expression data from the Cancer Genome Atlas (TCGA) database, to analyze the expression of *BAK1*, *NLRP1*, *CHMP7*, and *RIPK1* genes (Tang et al., 2017). The Cancer Genome Atlas (GSCA) (http://bioinfo.life.hust.edu.cn/web/GSCALite/) was also used to analyze target gene expression in tumors (Liu et al., 2018). UALCAN (http://ualcan.path.uab.edu) analyzes cancer and paracancer gene expression data in depth using TCGA data (Chandrashekar et al., 2017). In addition, it can be used to analyze the correlation between gene expression and clinical information, including age, gender, tumor clinical staging, tumor pathological staging, and other clinical data.

### Mutation and correlation analysis of *BAK1*, *NLRP1*, *CHMP7*, and *RIPK1* genes

CbioPortal (cBio Cancer Genomics Portal) (http://www.cbioportal.org/) was used to study gene mutation information in tumors, and we identified *BAK1*, *NLRP1*, *CHMP7*, and *RIPK1* gene mutations in HNSCC using the TCGA-HNSCC status (Gao et al., 2013). Meanwhile, GeneMANIA (http://genemania.org/), a website for constructing gene networks and functional prediction, was used to study the interaction of *BAK1*, *NLRP1*, *CHMP7*, and *RIPK1* genes (Warde-Farley et al., 2010).

### Survival analysis

We used the kaplan-meier plotter (http://kmplot.com/analysis/) to analyze the survival curves of different genes in HNSCC, where we chose the best cutoff value for the selection, where higher than this value is considered high expression and lower than this value is low expression, where the vertical lines are censored data (Lánczky and Győrffy, 2021). In addition to overall survival (OS), we analyzed Disease Free Survival (DFS), progression-free interval (PFI), and progression-free interval survival data.

### Combined indicator long non-coding RNAs (lncRNAs) receiver operator characteristic (ROC) curves for NPC diagnosis

The diagnostic effectiveness and diagnostic value of single or multiple combined indicators (biomarkers) were determined using ROC curves. The pROC package (R4. 0.3 software) was used to conduct the analysis. The area under the curve (AUC) represents the clinical significance of the experiment. Generally, an AUC value closer to 1.0 indicates high accuracy, and vice versa. Multiple lncRNAs (identified using the preceding procedures) were subjected to the process of single or multiple combined-indicator ROC curve analysis. The pROC package in the R4. 0.3 software was used for analysis and determining diagnostic values.

### Enrichment analysis of NPC-related PAGs

The ROC analysis method was used on 52 PAGs to screen genes with AUC values >0.5. As in previous studies, the “clusterProfiler” and “org. Hs. eg. db” packages (R 4.0.3 software) were used for the Kyoto Encyclopedia of Genes and Genomes (KEGG) pathway and Gene Ontology (GO) enrichment analyses (Lin et al., 2021; Wu et al., 2021; Zhao and Jiang, 2022). In addition, the “clusterProfiler” package was used to analyze and visualize the genes and gene clusters in functional profiles (GO and KEGG). Biological process (BP), molecular function (MF), and cellular component (CC) are the three components associated with GO analyses (screening criteria: Q-value < 0. 05; *p*-value < 0. 05).

We analyzed the functional enrichment of *BAK1*, *NLRP1*, *CHMP7*, and *RIPK1* genes in the TCGA-HNSCC status and applied the LinkedOmics database (http://www.linkedomics.org/login.php) for analysis. We chose TCGA_HNSCC and RNA-seq data on this website and entered *BAK1*, *NLRP1*, *CHMP7*, and *RIPK1* genes using the Pearson Correlation test. Later, we selected over-representation analysis (ORA) as an enrichment tool, PANTHER pathway data as functional data, and rank criteria as *p*-value, with <0.05 being considered statistically significant (Vasaikar et al., 2018).

### Immunomodulator analysis

We used the TISIDB database (http://cis.hku.hk/TISIDB/index.php) to analyze the correlation between genes and immunomodulation-related genes in HNSCC, where immunosuppressive markers included CD244, CD274, CTLA4, and LGALS9, the immune activation marker was ICOS, and major histocompatibility complex (MHC) molecules included HLA-E (Ru et al., 2019).

### Immuno-infiltration analysis

For immune infiltration analysis, we used the tumor immune estimation resource (TIMER) database (https://cistrome.shinyapps.io/timer/). Immune cells were selected as B cells, CD8^+^ T cells, CD4^+^ T cells, macrophages, neutrophils, and dendritic cells (DCs). The correlation between the aforementioned immune cells and the genes *BAK1*, *NLRP1*, *CHMP7*, and *RIPK1* was analyzed (Li et al., 2016; Li et al., 2017). Subsequently, we used the TIMER database to analyze the correlation between the degrees of immune cell infiltration of HNSCC tumors and the variation in the copy number of different somatic cells of the gene.

### Construction of the prognostic model

The survival analyses method was used to screen the genes associated with the prognosis of patients with HNSCC. The gene expressions were combined with the clinical prognostic information of the patients. After the false discovery rate (FDR) is corrected for both univariate Cox regression analysis and Kaplan-Meier (KM) survival analysis results, we identified the other genes that affected the prognosis of HNSCC patients using the univariate Cox regression analysis method (criterion: *p* < 0. 05). Genes were used as dependent variables for curve fitting to obtain an optimal Cox proportional risk regression model. The model characterized by the minimum Akaike information criterion (AIC) value was selected. The low AIC value indicated that the model contained few free parameters and could be used to analyze the data efficiently.

### Validation of the prognostic model

Following the identification of the optimal model, the risk scores were calculated. The risk scores were analyzed, and based on the risk scores at the maximum of the Youden index in the ROC curve, the patients were classified into low- and high-risk groups. The following methods were used to determine whether the risk scores could influence a patient’s prognosis for hepatocellular carcinoma (Bray et al., 2018): non-parametric tests were conducted to compare the differences in risk scores by studying various clinicopathological factors (sex, age, pathological stage, clinical stage, grading, and TNM stage) (Ferlay et al., 2019); the survival curves of the prediction model for patients with HNSCC were plotted using the survival analysis method (Johnson et al., 2020); the prediction accuracy was studied by analyzing the time-dependent ROC curves generated using R software; and (Stein et al., 2015) Cox regression analysis results were used to determine if the risk score and other clinicopathological factors contributed to patients’ poor prognosis for HNSCC.

### Nomogram construction and calibration curve plotting

A nomogram was generated with the “rms” package in R, and the calibration curves were plotted for 1-, 3-, and 5-years OS. The risk score, sex, age, grading, clinical stage, and tumor stage were analyzed to obtain the results. Additionally, the Hosmer-Lemeshow test was employed to check whether the predicted and actual outcomes agreed.

### Analysis of the level of immune cell infiltration and the tumor microenvironment

Various analytical methods for detecting immune cell infiltration are currently available. These methods include TIMER, CIBERSORT, XCELL, QUANTISEQ, McCounter, EPIC, and CIBERSORT on TIMER2 (Newman et al., 2015; Becht et al., 2016; Aran et al., 2017; Li et al., 2017; Racle et al., 2017; Chen et al., 2018; Finotello et al., 2019; Deng et al., 2021; Mei et al., 2022; Sun et al., 2022). The correlation coefficients for the correlation between different risk scores (obtained using different calculation methods) and certain immune cells can be obtained by determining the relationship between the immune cells and risk scores. The R software packages limma, ggplot2, scales, ggtext, ggpubr, and tidyverse were used for analysis, and the results were visualized using bubble plots. The scores for immune cells and immune-related functions were obtained using the single-sample gene set enrichment analysis (ssGSEA) technique. Additionally, using limma, ggpubr, and reshape2 (R software) were used to determine the differences between the immune cells and immune-related functions corresponding to the low- and high-risk groups. Finally, the TME was scored using the estimate package (R software) to compare the TME between the two groups.

### Statistical analysis

Statistical results were presented as mean ± standard deviation, and statistical differences between the two samples were analyzed using two-tailed t-tests or analysis of variance. *p*-value ≤ 0.05 was considered statistically significant.

## Results

### Data download and pre-processing

Three NPC datasets (GSE12452, GSE53819, and GSE64634) were downloaded from the Gene Expression Omnibus (GEO) database, normalized, and batch corrected to form a dataset with 61 NPC samples and 32 normal samples (16,820 genes). The TCGA database was analyzed, and the transcript data and relevant clinical information for HNSCC patients (*n* = 360) were downloaded from it.

### Pyroptosis-related lncRNAs ROC curves for NPC diagnosis

**FIGURE 1:**
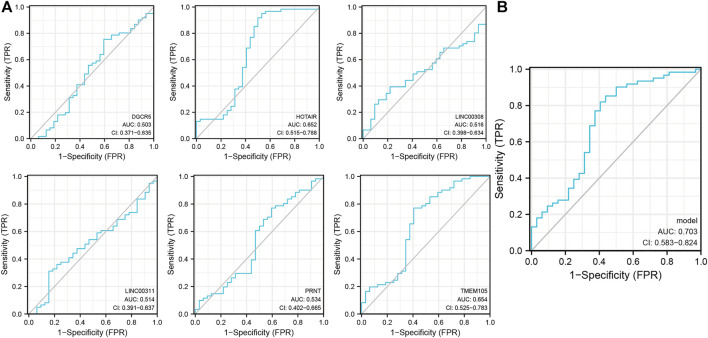
Single- and combined-indicator receiver operating characteristic curve (ROC) curves of pyroptosis-related long non-coding RNA (lncRNAs) for nasopharyngeal carcinoma (NPC) diagnosis. **(A)**. Single-indicator ROC curves for *DGCR5*, *HOTAIR*, *LINC00308*, *LINC00311*, *PRNT*, and *TMEM105* for NPC diagnosis; **(B)**. Six lncRNAs as co-diagnostic biomarkers.

The raw dataset included 15,153 messenger RNA (mRNAs) and 199 lncRNAs. Previously reported results were analyzed to extract 52 PAGs to obtain the relevant expression profile (Supplementary Table 1). Subsequently, six pyroptosis-related lncRNAs (*DGCR5*, *HOTAIR*, *LINC00308*, *LINC00311*, *PRNT*, and *TMEM105*) were identified (*p* < 0. 001; correlation coefficient ≥0. 4) using three NPC datasets (GSE12452, GSE53819, and GSE64634). The lncRNAs were analyzed using the single-indicator ROC curve analysis method. The following results have been presented: DGCR5: (AUC = 0.503, 95% confidence interval (CI): 0. 371–0. 635), HOTAIR: (AUC = 0.652, 95% CI: 0.515–0.788), LINC00308: (AUC = 0.516, 95% CI: 0.398–0.634), LINC00311: (AUC = 0.514, 95% CI: 0.391–0.637), PRNT: (AUC = 0.534, 95% CI: 0.402–0.665), and TMEM105: (AUC = 0.654, 95% CI: 0.525–0.783) (Figure 1A). Subsequently, the combined-indicator ROC curves for the six lncRNAs were plotted with an AUC of 0.703 and a 95% CI of 0.583–0.824 (Figure 1B). These results suggest that the six lncRNAs (DGCR5, HOTAIR, LINC00308, LINC00311, PRNT, and TMEM105) have good diagnostic values and can function as co-diagnostic biomarkers.

### Enrichment analysis of NPC-related PAGs

**FIGURE 2:**
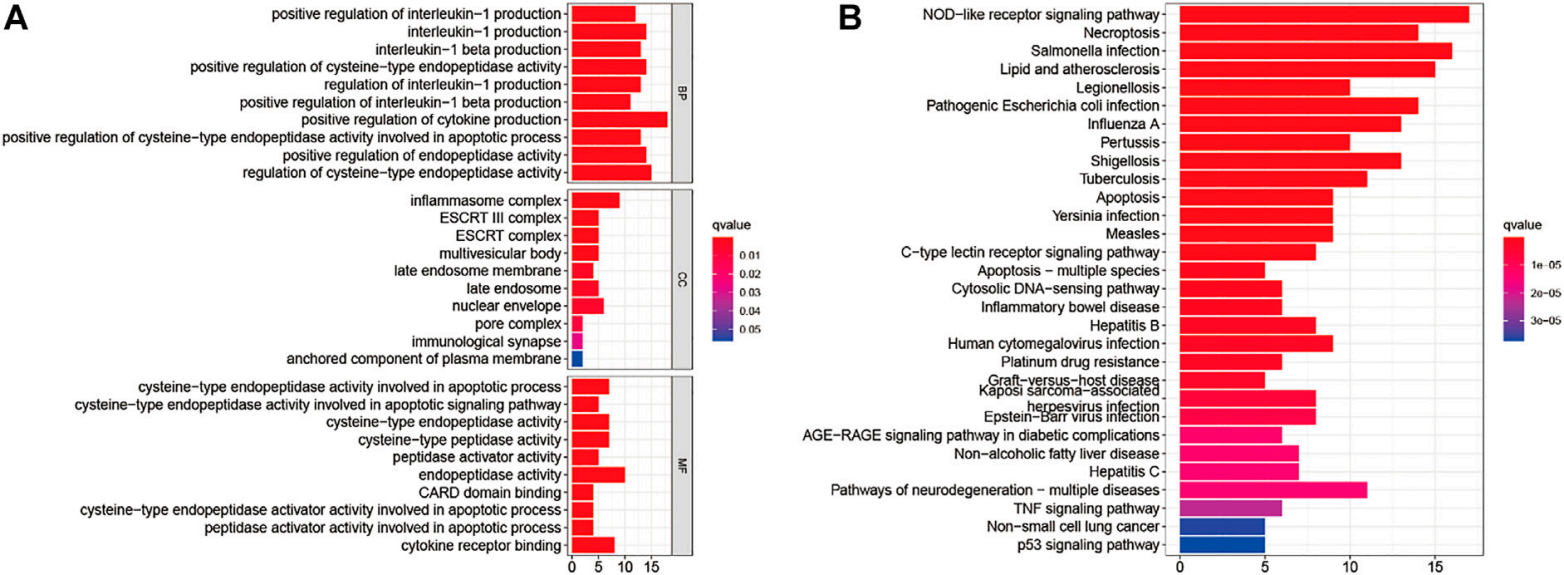
Gene Ontology **(A)** and The Kyoto Encyclopedia of Genes and Genomes **(B)** Enrichment analyses for nasopharyngeal carcinoma-related pyroptosis-associated genes.

Analyzing the ROCs yielded NPC-related 42 genes with AUC values >0.5 (*p*-value < 0.05; Q-value < 0.05; Supplementary Table S2). GO and KEGG analyses were performed on these genes. These 42 genes were enriched in BPs (positive regulation of cytokine production; positive regulation of cysteine-type endopeptidase activity involved in the apoptotic process; positive regulation of interleukin-1 production; and others); CCs (inflammasome complex, ESCRT III complex, multivesicular body, nuclear envelope, and others); and MFs (cysteine-type endopeptidase activity involved in the apoptotic signaling pathway; cysteine-type endopeptidase activator activity involved in apoptotic process; peptidase activator activity involved in apoptotic process; cytokine receptor binding; and others) (Figure 2A). The NOD-like receptor signaling pathway was the most enriched in KEGG pathways. *Salmonella* infection, necroptosis, lipid and atherosclerosis, legionellosis, pathogenic *Escherichia coli* infection, influenza A, pertussis, shigellosis, tuberculosis, apoptosis, *Yersinia* infection, measles, C-type lectin receptor signaling pathway, apoptosis-multiple species, cytosolic DNA-sensing pathway, inflammatory bowel disease, hepatitis B, human cytomegalovirus infection, platinum drug resistance, graft-versus-host disease, Kaposi sarcoma-associated herpesvirus infection, Epstein-Barr virus infection, AGE/RAGE signaling pathway in diabetic complications, non-alcoholic fatty liver disease, hepatitis C, pathways of neurodegeneration-multiple diseases, TNF signaling pathway, and non-small cell lung cancer p53 signaling pathway respectively (Figure 2B).

### Construction and evaluation of a prognostic model

**FIGURE 3:**
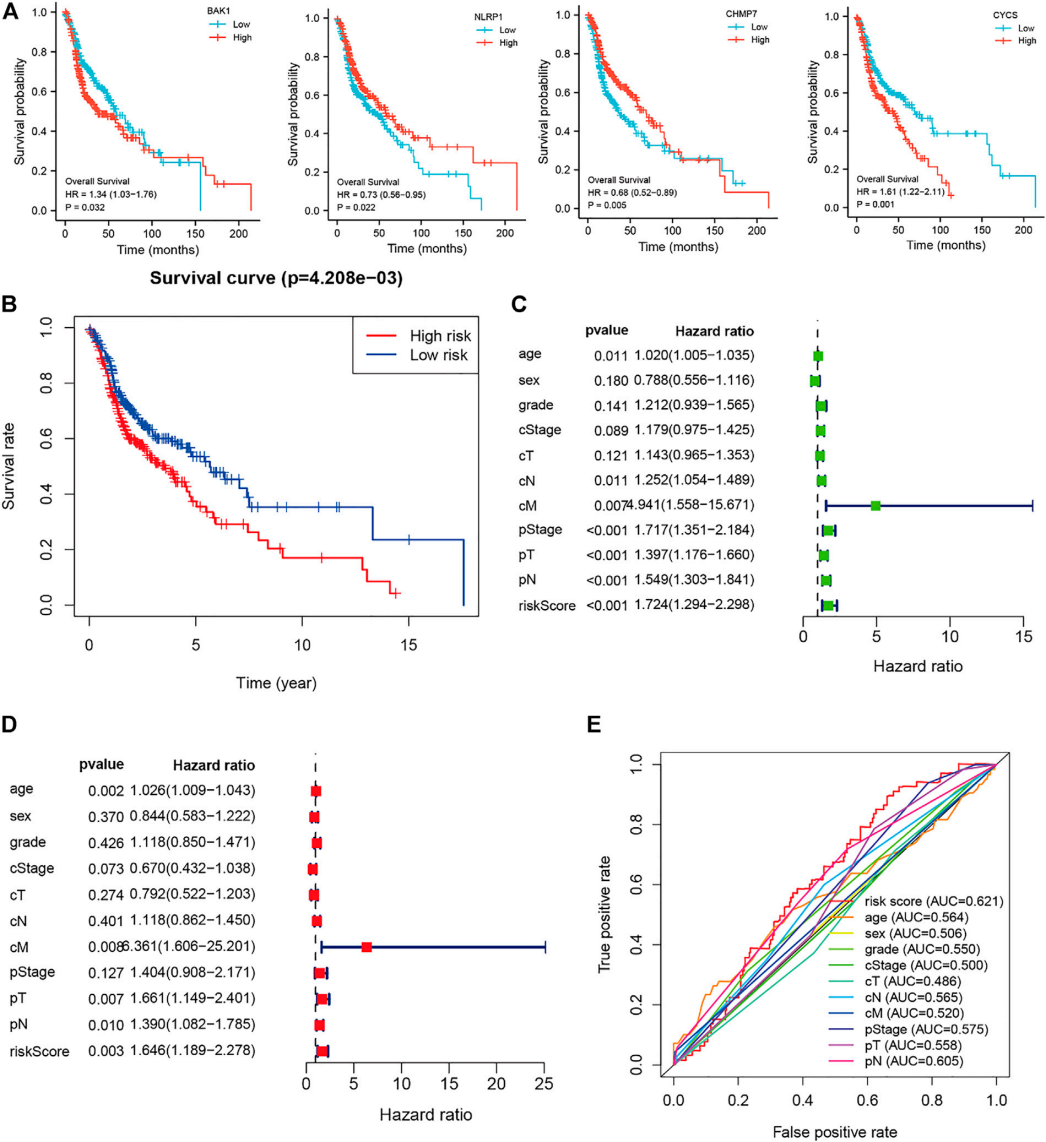
Risk and prognostic analysis of the single gene. **(A)**. Survival analysis to determine the correlation between the expressions of *BAK1*, *NLRP1*, *CHMP7*, and *CYCS* and head and neck squamous cell carcinoma (HNSCC); **(B)**. Kaplan-Meier survival curves present the correlation between the prognostic risk scores of patients suffering from HNSCC and the corresponding overall survival rates; **(C–D)**. The forest plot shows the univariate **(C)** and multivariate **(D)** Cox regression results; and **(E)**. Receiver operating characteristic curves are calculated for determining risk scores based on the sensitivity and specificity of the prognosis of patients with HNSCC.

**FIGURE 4:**
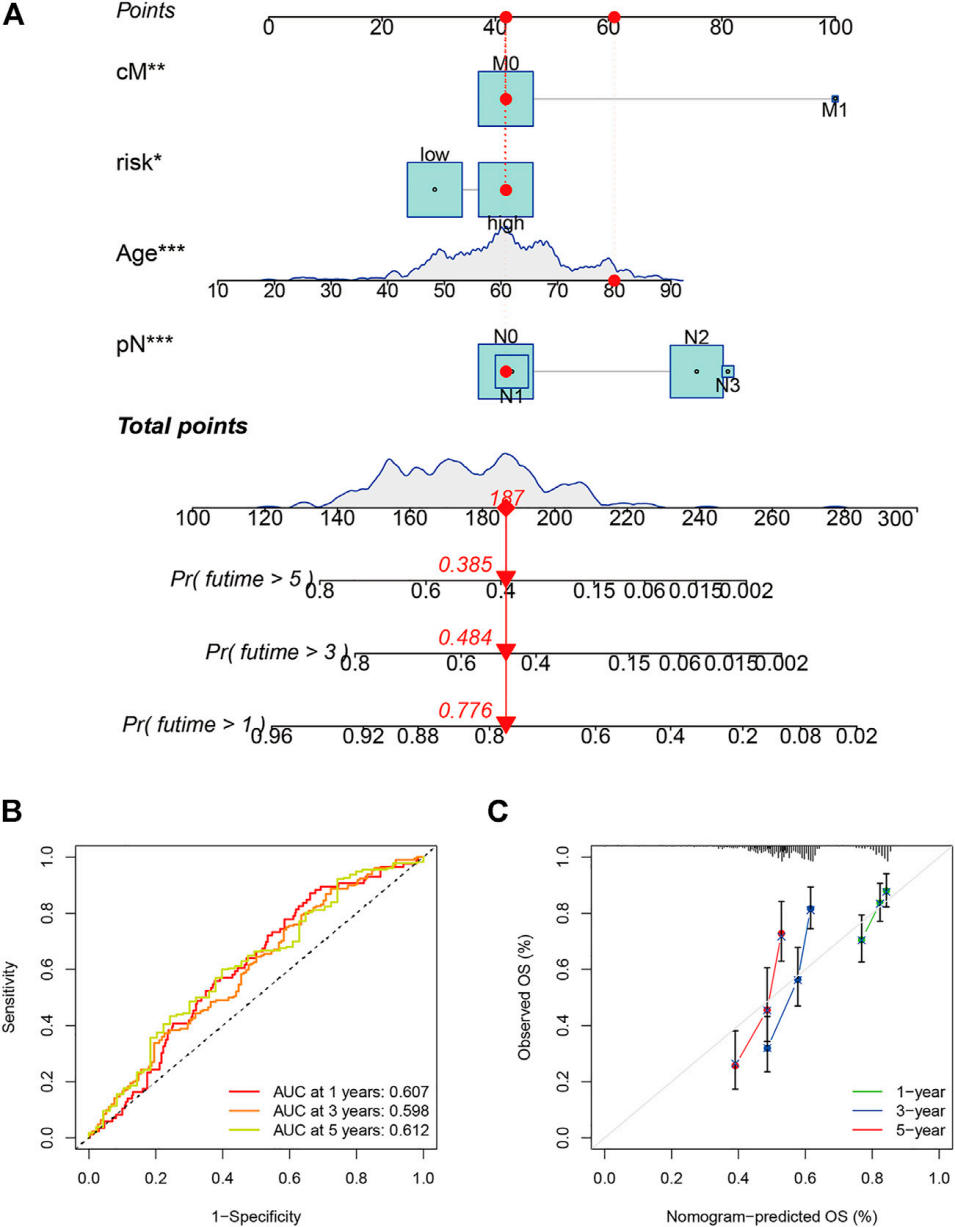
Evaluation and development of the prognostic model. **(A)** Prognostic model (nomogram) constructed using the “rms” R package; **(B)** The area under the curves for 1-, 3-, and 5-years clinical outcomes (0.607, 0.598, and 0.612, respectively). The values indicate good predictive power; and **(C)** Calibration plots reflect the agreement between the predicted and actual survival rates.

The 42 PAGs were incorporated into the TCGA database and four genes [*BAK1* (*p* = 0.032, HR = 1.34 (1.03–1.76)], *NLRP1* [*p* = 0.022, HR = 0.73 (0.56–0.95)], *CHMP7* [*p* = 0.005, HR = 0.68 (0.52–0.89)], and *CYCS* [*p* = 0.001, HR = 1.61 (1.22–2.11)] associated with the prognosis of HNSCC patients were identified following the process of survival analysis (Figure 3A). Subsequently, the minimum AUC value was considered when selecting the best model, which consisted of three genes (*NLRP, CHMP7*, and *CYCS*). The risk score was calculated as follows: risk score = NLRP1*(−0. 067) + CHMP7*(−0. 044) + CYCS*(0. 111). The patients were then classified into two groups (low-risk and high-risk) based on their risk scores. The median risk score was used as the cut-off value. Analyzing the K-M survival curves showed that the OS of patients in the high-risk group was lower than that of patients in the low-risk group (*p* = 4.208e-03) (Figure 3B). This indicated that the prognosis could be predicted using the risk scores. The multivariate and univariate Cox regression analysis method was used to assess the clinical prognostic factors (sex, age, stage, pathological TNM staging, and clinical TNM staging) and risk scores to determine whether the survival model could function as an independent prognostic factor for HNSCC. The HR values for the risk scores obtained using the multivariate and univariate Cox regression analyses were 1.646 (95% CI: 1.189–2.278, *p* = 0.003) and 1. 724 (95% CI: 1.294–2.298, *p* < 0.001), respectively (Figures 3C,D). This indicated that the risk model could be used as an independent prognostic factor for HNSCC. Furthermore, the AUC value (0.621) for the risk score was calculated to assess its predictive sensitivity and specificity (Figure 3E). The findings suggested that the developed risk model was a viable independent prognostic factor for HNSCC patients. Moreover, to predict patient prognosis, “rms” (an R package) was used to construct a nomogram based on risk score, age, sex, grade, clinical stage, and tumor stage (Figure 4A). The survival rate for each individual was calculated using the total score obtained by adding all the scores corresponding to each variable. The process was used to obtain the 1-, 3-, and 5-years OS. The corresponding AUCs were 0.607, 0.598, and 0.612, indicating good predictive performance (Figure 4B). The analysis of nomogram calibration plots revealed that the predicted survival rate was in good agreement with the actual survival rate (Figure 4C).

### Expression of *BAK1, NLRP1, CHMP7*, and *RIPK1* in HNSCC and normal tissues

**FIGURE 5:**
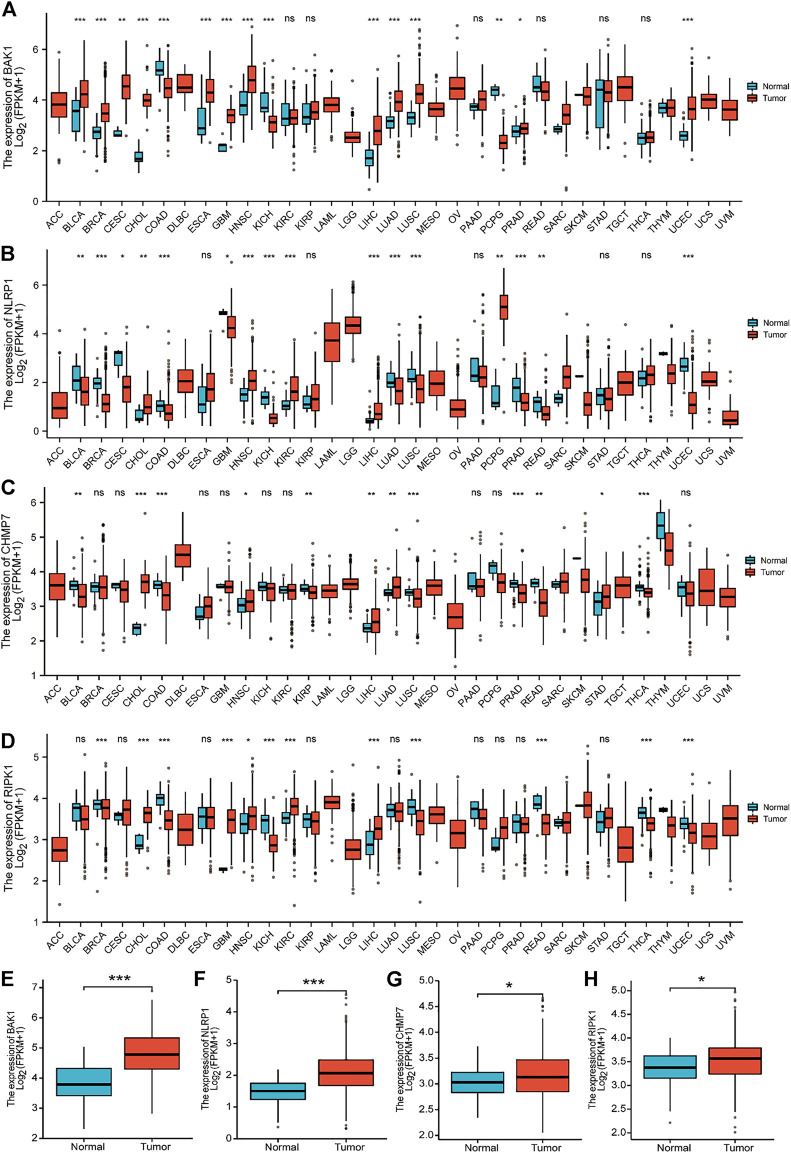
Expression of *BAK1*, *NLRP1*, *CHMP7*, and *RIPK1* markers in tumors. **(A)** BAK1 expression in 33 tumor species in the Cancer Genome Atlas (TCGA) database; **(B)** Expression of NLRP1 in tumor of 33 species in the TCGA database; **(C)** CHMP7 expression in 33 tumor species in the TCGA database; **(D)** TCGA database of RIPK1 expression in 33 tumor species; **(E)** BAK1 expression is increased in head and neck squamous cell carcinoma (HNSCC) tissues; **(F)** NLRP1 expression is elevated in HNSCC tissues; **(G)** CHMP7 expression is elevated in HNSCC tissues; **(H)** RIPK1 expression was elevated in HNSCC tissues. *p < 0.05, **p < 0.01, ***p < 0.001.

Figures 5A–D show the expression of BAK1, NLRP1, CHMP7, and RIPK1 markers in 33 tumors. These four markers were overexpressed in cholangiocarcinoma (CHOL), HNSCC, and liver hepatocellular carcinoma (LIHC), and they were all statistically different. Figures 5E–H show that the expression of BAK1, NLRP1, CHMP7, and RIPK1 was higher in HNSCC tumor tissues than in paraneoplastic tissues.

### Mutation of *BAK1*, *NLRP1*, *CHMP7*, and *RIPK1* genes in HNSCC and gene interaction network

**FIGURE 6:**
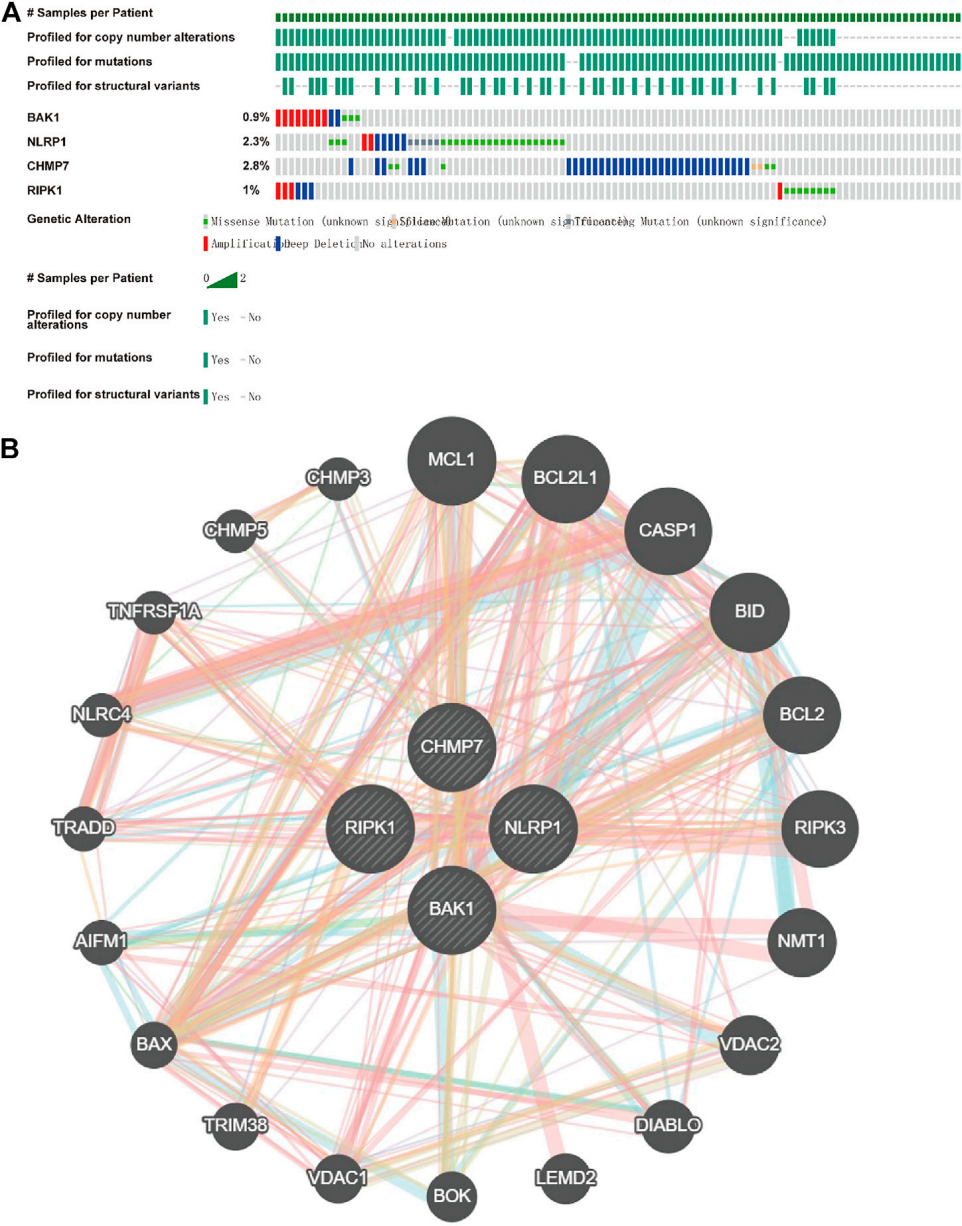
Mutations of *BAK1*, *NLRP1*, *CHMP7*, and *RIPK1* genes in head and neck squamous cell carcinoma (HNSCC) and their interplay network. **(A)** Total mutations of *BAK1, NLRP1, CHMP7,* and *RIPK1* in HNSCC; **(B)** GeneMANIA demonstrates the gene interaction network of *BAK1*, *NLRP1*, *CHMP7*, and *RIPK1*.

Because all the above genes were expressed at higher levels in HNSCC, we investigated their mutations in HNSCC using cBioportal and found that *BAK1*, *NLRP1*, *CHMP7*, and *RIPK1* genes were highly conserved in HNSCC (Figure 6A). The interaction of the genes above was then investigated using GeneMANIA (http://genemania.org). The interplay network of the *BAK1*, *NLRP1*, *CHMP7*, and *RIPK1* genes may include 20 potential target genes (Figure 6B).

### Prognostic role of *BAK1*, *NLRP1*, *CHMP7*, and *RIPK1* in HNSCC

**FIGURE 7:**
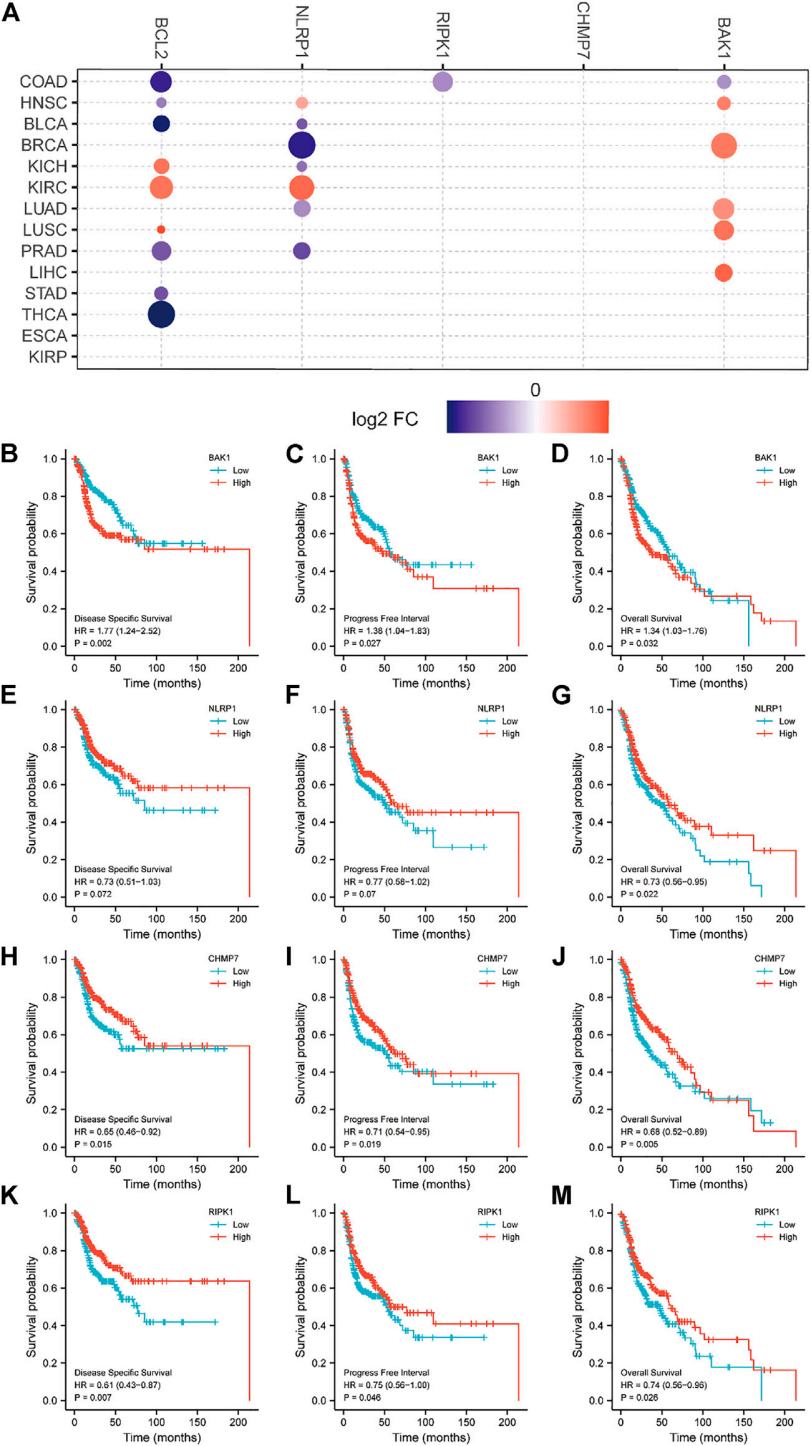
Relationship between BAK1, NLRP1, CHMP7, and RIPK1 messenger RNAs (mRNAs) and prognosis of head and neck squamous cell carcinoma (HNSCC) patients. **(A)** Relationship between BAK1, NLRP1, CHMP7, and RIPK1, Bcl-2 mRNAs expression and tumor survival in the Cancer Genome Atlas database; Kaplan-Meier (K-M) plotter showing survival curves for disease free survival (DFS); **(B)** Progression-free interval (PFI); **(C)** Overall survival (OS); **(D)** For BAK1 and HNSCC; the K-M plotter shows DFS for NLRP1 and HNSCC; **(E)** PFI; **(F)** OS; **(G)** Survival plots; K-M plotter showing DFS; **(H)** PFI; **(I)** OS; **(J)** Survival plots for CHMP7 and HNSCC; K-M plotter showing DFS; **(K)** PFI; **(L)** OS; **(M)** Survival plots for RIPK1 and HNSCC.

We analyzed the relationship between *BAK1, NLRP1, CHMP7*, and *RIPK1* mRNA and HNSCC survival. Figure 7A demonstrates the relationship between *BAK1, NLRP1, CHMP7*, and *RIPK1* mRNAs and tumor survival. The Kaplan-Meier (K-M) plotter was also used to analyze its relationship with the prognosis of HNSCC patients. As shown in Figures 7B–D, the higher expression of *BAK1* worsens the HNSCC prognosis. Unlike *BAK1*, the higher expression of *NLRP1, CHMP7*, and *RIPK1* improves HNSCC prognosis, as shown in Figures 7E–M.

### Relationship between *BAK1, NLRP1, CHMP7*, and *RIPK1* mRNAs and clinical characteristics in HNSCC

Because the above results showed that *BAK1, NLRP1, CHMP7*, and *RIPK1* mRNAs and HNSCC prognosis were closely related, we investigated the relationship between the above genes and the clinical characteristics of HNSCC further. Table 1 shows that in HNSCC, BAK1 and CHMP7 are associated with sex, clinical staging, and tumor histological grading, NLRP1 is associated with sex and clinical staging, and RIPK1 is not associated with any of these clinical characteristics.

**TABLE 1:** Demographic characteristics of the patients.

Variable	Total
Age	60.84 ± 11.85
Sex	
Male	264
Female	96
Grade	
G1	43
G2	229
G3	87
G4	1
cStage	
Stage I	16
Stage II	60
Stage III	81
Stage IV	203
cT	
T1	27
T2	85
T3	96
T4	152
cN	
N0	183
N1	69
N2	103
N3	5
cM	
M0	356
M1	4
pSatge	
Stage I	20
Stage II	46
Stage III	64
Stage IV	230
pT	
T1	31
T2	94
T3	82
T4	153
pN	
N0	154
N1	56
N2	143
N3	7

### Functional analysis of the genes *BAK1*, *NLRP1*, *CHMP7*, and *RIPK1* in HNSCC

**FIGURE 8:**
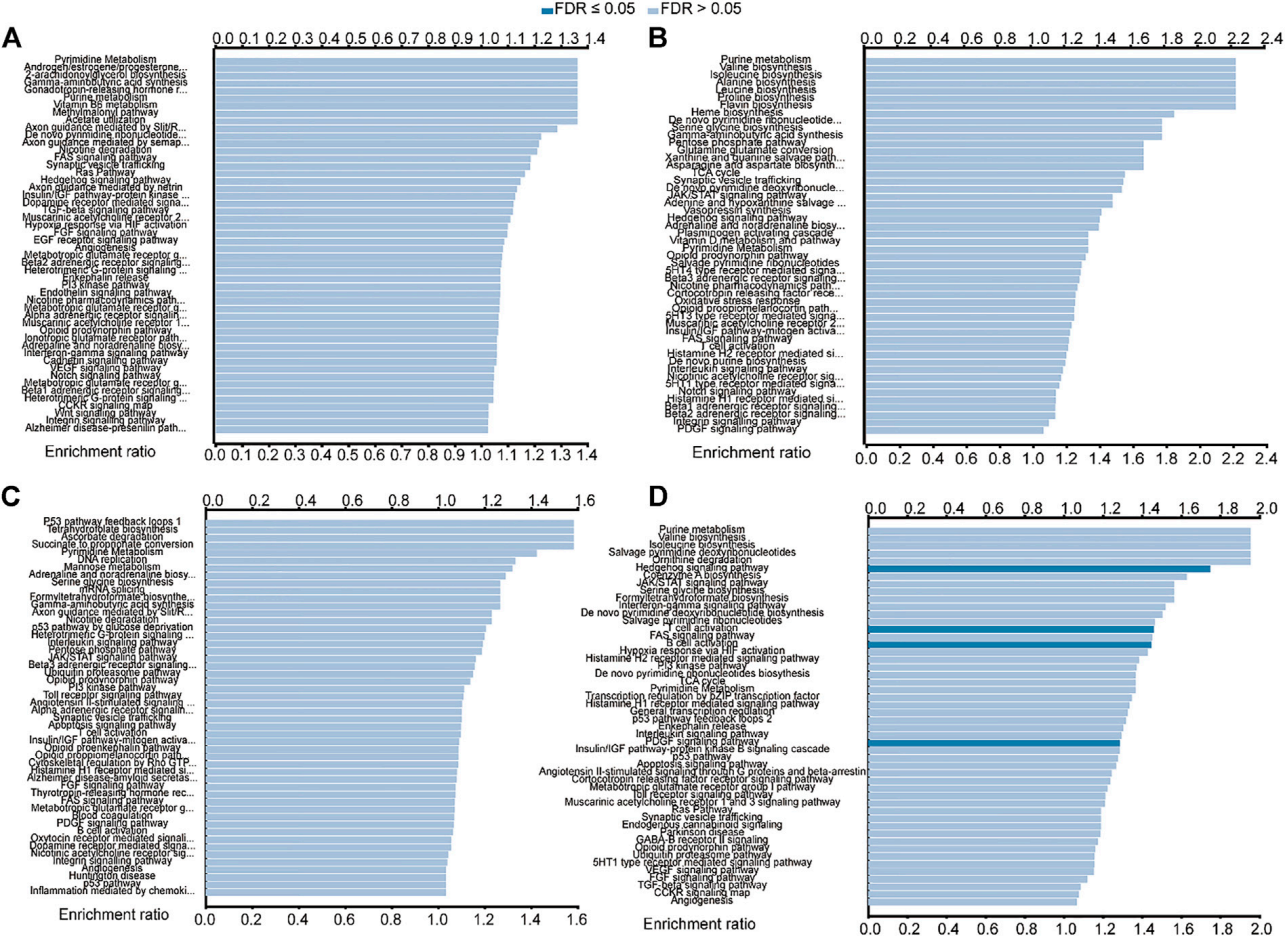
Functional enrichment analysis of *BAK1*, NLRP1, CHMP7, and RIPK1 in head and neck squamous cell carcinoma (HNSCC) from the LinkedOmics database. **(A)** Functional enrichment analysis of *BAK1* in HNSCC; **(B)** Functional enrichment analysis of NLRP1 in HNSCC; and **(C)** Functional enrichment analysis of CHMP7 in HNSCC; and **(D)** Functional enrichment analysis of RIPK1 in HNSCC.

The LinkedOmics database was used to further analyze the predicted function of the above genes in HNSCC. As shown in Figure 8A, *BAK1* is primarily enriched in pathways such as pyrimidine metabolism, androgen/estrogen/progesterone biosynthesis, 2-arachidonoylglycerol biosynthesis, and gamma-aminobutyric acid synthesis. As shown in Figure 8B, *NLRP1* functions are mainly enriched in purine metabolism, valine biosynthesis, isoleucine biosynthesis, and alanine synthesis. As shown in Figure 8C, the functions of *CHMP7* are primarily enriched in pathways such as P53 pathway feedback loops, tetrahydrofolate biosynthesis, ascorbate degradation, and succinate to propionate conversion. Similarly, the functions of *RIPK1* are mainly enriched in pathways such as purine metabolism, valine biosynthesis, isoleucine biosynthesis, and salvage pyrimidine deoxyribonucleotides, as shown in Figure 8D.

### Analysis of immune cell infiltration levels and TME

**FIGURE 9:**
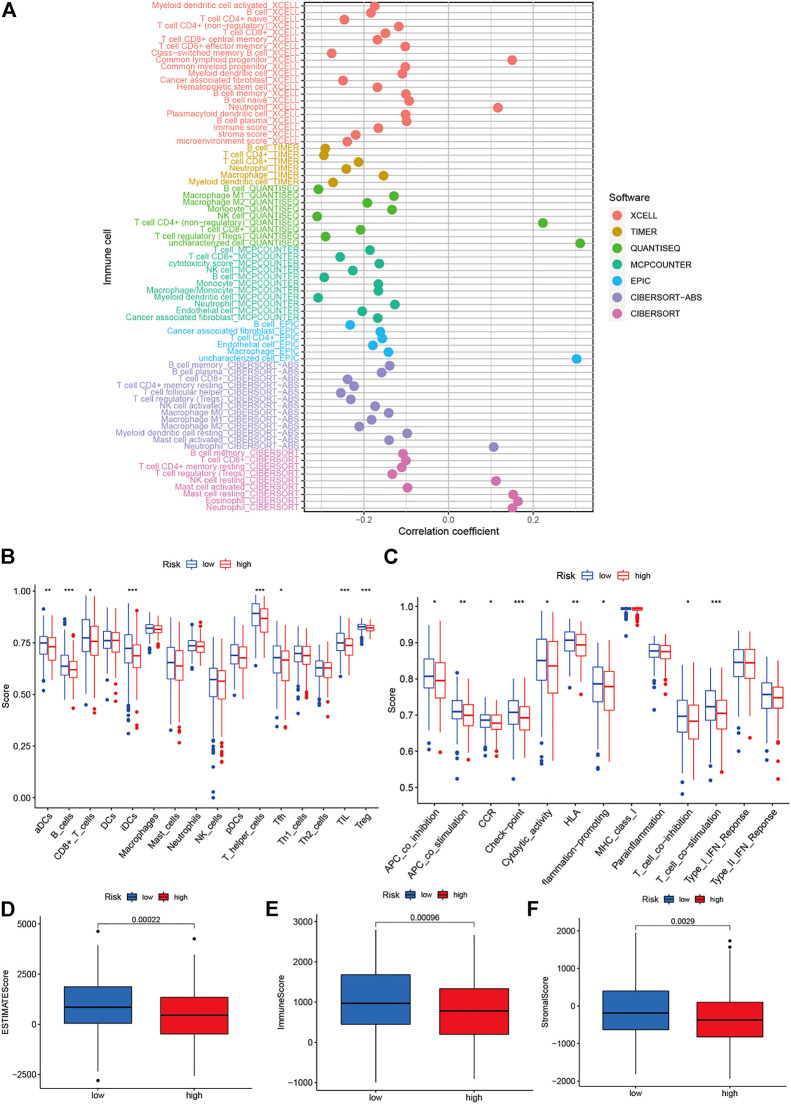
Analysis of tumor microenvironment and of degrees mmune cell infiltration. **(A)** Determination of the relationship between immune cells and risk scores using different calculation methods; **(B–C)** Immune cell and immune-related function scores were obtained using the single-sample gene set enrichment analysis technique; and **(D–F)** The differences between the immune cell infiltration levels in the low- and high-risk groups.

The immune cells and risk scores were correlated using different calculation methods. The findings suggested a correlation between the immune cells and low-risk populations. The low-risk population, in particular, was associated with the CD4^+^ T cells, immune score, and DCs using the XCELL algorithm; CD4^+^ T cells and CD8^+^ T cells using the TIMER algorithm; macrophages using the QUANTISEQ algorithm; CD8^+^ T cells using the MCPCOUNTER algorithm; B cells using the EPIC algorithm; CD4^+^ T cells, B cells, and CD8^+^ T cells using the CIBERSORT-ABS algorithm; and CD8^+^ T and B cells using the CIBERSORT algorithm (Figure 9A). Moreover, the ssGSEA scores for immune cells and immune-related functions revealed a difference in the distribution patterns of Th cells, regulatory T cells, tumor-infiltrating lymphocytes, follicular helper T cells, adipose-derived cells, interdigitating dendritic cells, CD8^+^ T cells, and B cells between the high- and low-risk groups. Furthermore, a difference was observed in the immune functions (for the antigen-presenting cells, T cells, and immune checkpoints) associated with the two groups (Figures 9B,C). The TMEs corresponding to the low- and high-risk groups were analyzed, and the results indicated that the immune scores of the high-risk patients were lower than those of the low-risk patients (Figures 9D–F).

### Correlation between *BAK1*, *NLRP1*, *CHMP7*, and *RIPK1* genes and immunomodulators

**FIGURE 10:**
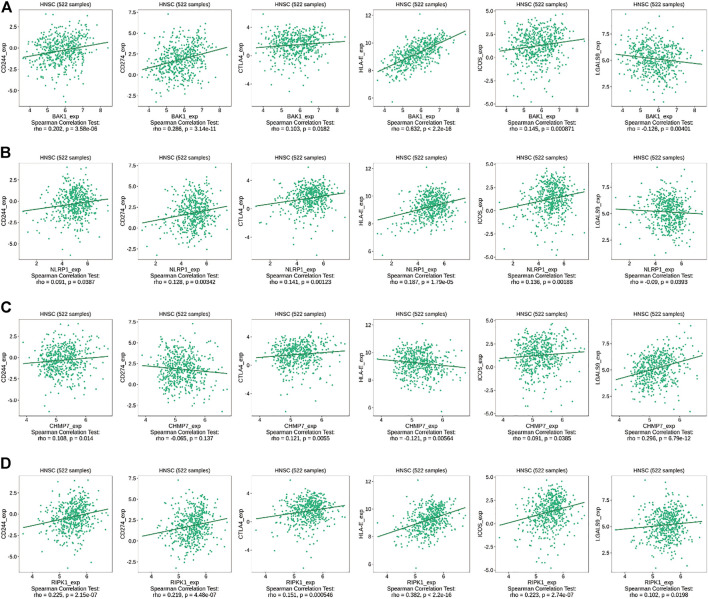
Correlation between *BAK1*, *NLRP1*, *CHMP7*, and *RIPK1* genes and immunomodulators. **(A)** Correlation between *BAK1* gene and CD244, CD274, CTLA4, HLA-E, ICOS, and LGALS9 in HNSCC according to TISIDB database; **(B)** Correlation between *NLRP1* gene and CD244, CD274, CTLA4, HLA-E, ICOS, and LGALS9 in HNSCC according to TISIDB database; **(C)** Correlations between *CHMP7* gene and CD244, CD274, CTLA4, HLA-E, ICOS, and LGALS9 in HNSCC according to TISIDB database; and **(D)** Correlations between *RIPK1* gene and CD244, CD274, CTLA4, HLA-E, ICOS, and LGALS9 in HNSCC according to TISIDB database.

Interaction network results revealed that *BAK1*, *NLRP1*, *CHMP7*, and *RIPK1* genes could interact with immune factors, including TNF-α, suggesting their influence on the immune microenvironment of the HNSCC tumor. We used the TISIDB database to investigate the relationship between the above genes and immunomodulators. As shown in Figure 10A, in HNSCC, *BAK1* has a positive correlation with CD244, CD274, CTLA4, HLA-E, and ICOS, while having a negative correlation with LGALS9; as shown in Figure 10B, *NLRP1* has a positive correlation with CD244, CD274, CTLA4, HLA-E, and ICOS, while having a negative correlation with LGALS9; as shown in Figure 10C, *CHMP7* correlates positively with CD244, CTLA4, ICOS, and LGALS9, and negatively with CD274 and HLA-E; similarly, as shown in Figure 10D, *RIPK1* correlates positively with CD244, CD274, CTLA4, HLA-E, ICOS, and LGALS9.

### Correlation of *BAK1*, *NLRP1*, *CHMP7*, and *RIPK1* genes’ expression and immune infiltration

**FIGURE 11:**
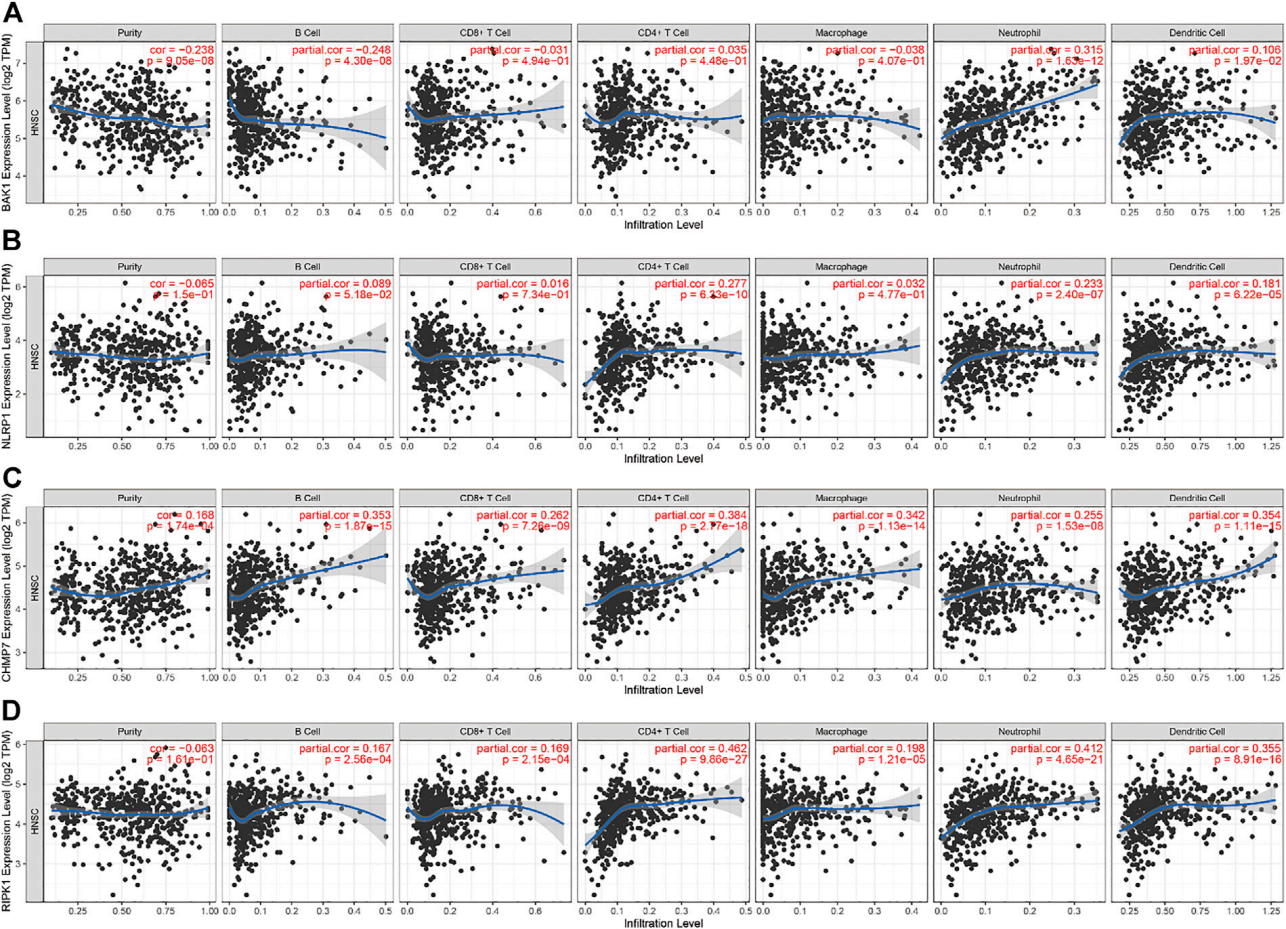
Correlation between *BAK1*, *NLRP1*, *CHMP7*, and *RIPK1* genes’ expression and immune infiltration. **(A)** Correlation between *BAK1* expression levels and B cell, CD8^+^ T cell, CD4^+^ T cell, macrophage, neutrophil, dendritic cell (DC) infiltration; **(B)** Correlation between *NLRP1* expression levels and B cells, CD8^+^ T cells, CD4^+^ T cells, macrophages, neutrophils, and DC infiltration; **(C)** Correlation between *CHMP7* expression levels and the infiltration of B cells, CD8^+^ T cells, CD4^+^ T cells, macrophages, neutrophils, and DCs; and **(D)** Correlation between *RIPK1* expression levels and the infiltration of B cells, CD8^+^ T cells, CD4^+^ T cells, macrophages, neutrophils, and DCs.

In HNSCC, the expression of *BAK1*, NLRP1, CHMP7, and RIPK1 genes and numerous immunomodulatory markers showed a certain relationship. We used the TIMER database to analyze the relationship between different genes and immune infiltration. We selected B cells, CD8^+^ T cells, CD4^+^ T cells, macrophages, neutrophils, and DCs for immune cell infiltration analysis. As shown in Figure 11A, in HNSCC, *BAK1* has a positive correlation with CD8^+^ T cells, CD4^+^ T cells, neutrophils, and DC infiltration while having a negative correlation with B cells and macrophage cell infiltration; as shown in Figures 11B–D, *NLRP1*, *CHMP7,* and *RIPK1* genes and immune cell infiltration all show some positive correlation.

**FIGURE 12:**
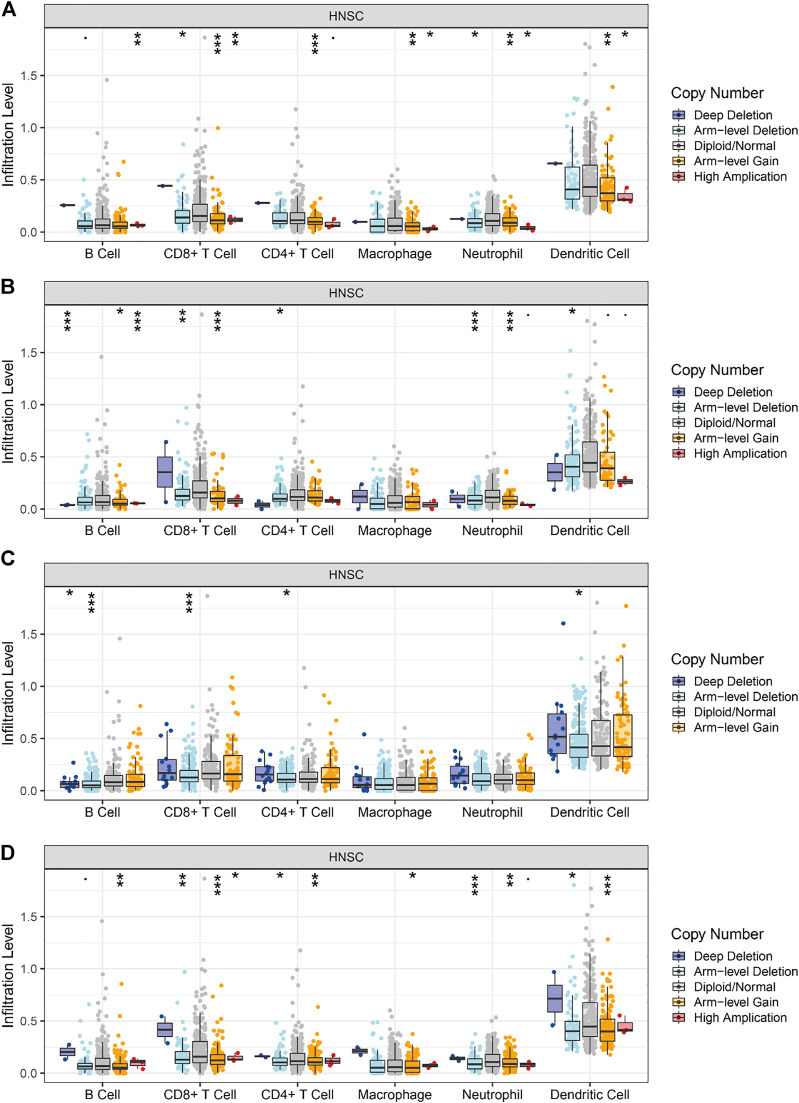
Correlation of *BAK1*, *NLRP1*, *CHMP7*, and *RIPK1* gene copy number variation and immune infiltration. **(A)** Correlation between *BAK1* gene copy number variation and B cell, CD8^+^ T cell, CD4^+^ T cell, macrophage, neutrophil, dendritic cell (DC) infiltration; **(B)** Correlation between *NLRP1* gene copy number variation and B cell, CD8^+^ T cell, CD4^+^ T cell, macrophage, neutrophil, DC infiltration; **(C)** Correlation between *CHMP7* gene copy number variation and B cell, CD8^+^ T cell, CD4^+^ T cell, macrophage, neutrophil, and DC infiltration; and **(D)** Correlation between *RIPK1* gene copy number variation and B cell, CD8^+^ T cell, CD4^+^ T cell, macrophage, neutrophil, and DC infiltration. *p < 0.05, **p < 0.01, ***p < 0.001.

All the above genes can influence tumor development by affecting immune infiltration. We used the TIMER database to analyze the correlation between the level of immune cell infiltration and gene copy number variation in HNSCC tumors. As shown in Figure 12A, the levels of B cell, CD8^+^ T cell, CD4^+^ T cell, macrophage, neutrophil, and DC infiltration decrease as the copy number of the *BAK1* gene increases. As shown in Figure 12B, the levels of B cell, CD4^+^ T cell, and DC infiltration increase as the copy number of the *NLRP1* gene increases, whereas the levels of CD8^+^ T cell, neutrophil, and macrophage decrease, and the changes in macrophage are not statistically significant. As shown in Figure 12C, when the *CHMP7* gene copy number is increased, B cell, CD8^+^ T cell, CD4^+^ T cell, and DC infiltration levels decrease; macrophages and neutrophils do not show statistical differences. As shown in Figure 12D, when the *RIPK1* gene copy number is increased, B cell, CD8^+^ T cell, CD4^+^ T cell, macrophage, neutrophil, and DC infiltration levels decrease.

In summary, the *BAK1*, *CHMP7*, and *RIPK1* genes can reduce immune cell infiltration by activating immunosuppressive markers, whereas *NLRP1* can both reduce immune infiltration by activating immunosuppressive markers and promote immune infiltration by activating immune activation markers.

## Discussion

HNSCC is a group of heterogeneous solid tumors originating from upper respiratory tract epithelial cells. It tends to metastasize and recur, increasing mortality, morbidity, and disability rates (Bray et al., 2018). Effective clinical risk assessment and early-stage diagnostic tools for HNSCC are scarce. Poor prognosis is primarily associated with local invasion, drug resistance, recurrence, and metastasis (Pulte and Brenner, 2010; Leemans et al., 2011). Traditional clinicopathological indicators, such as tumor size, vascular invasion, and TNM staging, cannot be used to stratify patients’ risks or predict their prognosis (Cheng et al., 2009). As a result, transcriptionomics and epigenetics should be used for screening potential biomarkers that aid in the early detection of the disease. The biomarkers have the potential for risk assessment, treatment, and monitoring of the prognosis of patients with HNSCC.

We obtained 52 PAGs by analyzing the NPC-related gene expression profile obtained from the GEO database. Six PAG-related lncRNAs were identified based on the correlation coefficient (r) and *p*-value (≥0.4 and <0.001, respectively). The single- and combined-indicator ROC curve analysis results suggested that the six lncRNAs had good diagnostic values and could be used as co-diagnostic biomarkers. These were accurate predictors of NPC. Moreover, 42 genes with AUC values of >0.5 were screened using the ROC analysis method. Their association with MF, CC, and BP was analyzed using the GO and KEGG enrichment analysis methods. Subsequently, the 42 genes were incorporated into the TCGA database, and the four genes (*BAK1*, *NLRP1*, *CHMP7*, and *CYCS*) associated with the prognosis of HNSCC patients were screened using the survival analysis method. The obtained genes were used as dependent variables for curve fitting to select the best Cox proportional risk regression model, which consisted of three genes (*NLRP1*, *CHMP7*, and *CYCS*). It has previously been reported that the genes incorporated into this model regulate tumor progression and significantly influence the process associated with the onset of oral squamous cell carcinoma, cutaneous squamous cell carcinoma, melanoma, breast cancer, and lung cancer (Sand et al., 2019; Xu et al., 2021).

NLRP1, a NOD-like receptor family protein, is widely expressed in various cell types. It is associated with the formation of inflammasomes. NLRP1 is linked to the production of IL-1 β and IL-18 and pyroptosis and plays a crucial role in developing innate immunity and generating inflammation. Thus, it influences the processes involved in the onset and progression of multiple diseases, including tumors, autoimmune diseases, neurological diseases, and metabolic diseases (Tupik et al., 2020). The downregulation of NLRP1 expression promotes the progression of human cutaneous squamous cell carcinoma (Sand et al., 2019). NLRP1 is also linked to the progression of various malignancies. It has also been reported that the regulation of TME by NLRP1 affects the prognosis of patients with lung adenocarcinoma (Shen et al., 2021). Moreover, elevated NLRP1 expression levels promote breast cancer cell proliferation, metastasis, and invasion. These processes are mediated by the induction of the process of epithelial-mesenchymal transition (Wei et al., 2017). The inflammasome is activated, and apoptotic pathways are inhibited in these conditions, resulting in the rapid progression of melanoma (Ehrhart et al., 1975).

As a component of the endosomal sorting complex (ESCRT III), CHMP7 significantly influences the processes of endosomal sorting, nuclear envelope formation, and neurodevelopment (Olmos et al., 2016; Sadoul et al., 2018). CHMP7 is also associated with the pathogenesis of amyotrophic lateral sclerosis. These conditions cause spinal cord damage, and bulbar muscular atrophy is observed under these conditions (Fairfield et al., 2019; Malik et al., 2019). A statistical relationship was found between CHMP7 expression levels and the clinical prognosis of cancer patients, and protein phosphorylation and immune cell infiltration processes were established (Guo et al., 2021).

CYCS is a central component of the mitochondrial electron transport chain. It is primarily associated with energy production in normal and tumor cells (Hüttemann et al., 2011). Mutations in this gene cause autosomal dominant thrombocytopenia, and apoptosis in oral squamous cell carcinoma cells is also triggered under these conditions (Ong et al., 2017; Uchiyama et al., 2018; Sabit et al., 2021).

To our knowledge, we are the first to report NPC-related PAGs. HNSCC was retrieved to screen the genes associated with the prognosis of HNSCC patients. A novel and robust prognosis assessment model for HNSCC patients has been developed. However, this study still has some limitations, and the sample size is small. Due to data set restrictions, there is a risk of racial bias in this study. In addition, bioinformatics analysis does not provide comprehensive results and should be supplemented with biological experiments. Further experimental studies are needed to gain a comprehensive understanding.

## Conclusion

In this study, NPC and PAGs were investigated in relation to nasopharyngeal carcinoma, and indicators related to the prognosis of HNSCC patients were identified. PAGs were used to develop and validate a prognostic model for NPC, and the genes incorporated into the model were closely related to the tumor microenvironment. Therefore, this study suggests that prognosis-related PAGs of NPC also predict the prognosis of HNSCC, which helps to improve our understanding of the treatment of NPC and HNSCC.

## Data Availability

Publicly available datasets were analyzed in this study. This data can be found here: The gene expression profile data related to nasopharyngeal carcinoma were obtained from GEO database (https://www.ncbi.nlm.nih.gov/geo/): GSE12452, GSE53819, and GSE64634. From TCGA database (https://www.cancer.gov/about-nci/organization/ccg/research), we downloaded the original RNA sequences and clinical data for HNSCC.
