# Supplementary material for: Development of a prognostic prediction model based on a combined multi-omics analysis of head and neck squamous cell carcinoma cell pyroptosis-related genes
Source: Front Genet. 2022 Sep 29;13:981222. doi: 10.3389/fgene.2022.981222 (PMC9557126; doi:10.3389/fgene.2022.981222)
Supplement: Supplementary file 3 [file Table1.DOCX]

Table 1. Demographic characteristics of the patients.

| Variable | Total |
| --- | --- |
| Age | 60.84±11.85 |
| sex | |
| Male | 264 |
| Female | 96 |
| Grade | |
| G1 | 43 |
| G2 | 229 |
| G3 | 87 |
| G4 | 1 |
| cStage | |
| Stage I | 16 |
| Stage II | 60 |
| Stage III | 81 |
| Stage IV | 203 |
| cT | |
| T1 | 27 |
| T2 | 85 |
| T3 | 96 |
| T4 | 152 |
| cN | |
| N0 | 183 |
| N1 | 69 |
| N2 | 103 |
| N3 | 5 |
| cM | |
| M0 | 356 |
| M1 | 4 |
| pSatge | |
| Stage I | 20 |
| Stage II | 46 |
| Stage III | 64 |
| Stage IV | 230 |
| pT |  |
| T1 | 31 |
| T2 | 94 |
| T3 | 82 |
| T4 | 153 |
| pN | |
| N0 | 154 |
| N1 | 56 |
| N2 | 143 |
| N3 | 7 |
